# Effect of accelerated high-fluence riboflavin and rose bengal-mediated corneal cross-linking on resistance to enzymatic digestion

**DOI:** 10.1186/s12886-024-03293-0

**Published:** 2024-01-24

**Authors:** Nikki L. Hafezi, M. Enes Aydemir, Nan-Ji Lu, Emilio A. Torres-Netto, Mark Hillen, Carina Koppen

**Affiliations:** 1 Faculty of Medicine, University of Antwerp, Wilrijk, Antwerp, Belgium; 2 ELZA Institute, Zurich, Switzerland; 3 Department of Ophthalmology, Antwerp University Hospital, Edegem, Antwerp, Belgium

**Keywords:** Corneal cross-linking, Collagenase, Cornea, Digestion, Riboflavin, Ultraviolet, Rose bengal, High fluence protocols, Accelerated CXL

## Abstract

**Purpose:**

This study evaluated the effect of high-fluence accelerated corneal cross-linking on the resistance to enzymatic digestion, assessing two chromophore/light combinations: riboflavin/UV-A light (RF/UV-A) and rose bengal/green light (RB/green).

**Methods:**

Freshly prepared ex-vivo porcine corneas (*n* = 189) were divided into 8 groups groups. Group A corneas were unirradiated controls without chromophore soaking (A0), or soaked with riboflavin (A1) or rose bengal (A2). Group B corneas underwent accelerated epi-off RF/UV-A CXL at fluences of 5.4 J/cm² (B1), 10 J/cm² (B2), or 15 J/cm² (B3). Group C corneas underwent accelerated epi-off RB/green CXL at fluences of either 10 J/cm² (C1) or 15 J/cm² (C2). Following CXL, all corneas were digested in 0.3% collagenase-A solution, and the time until complete dissolution was measured.

**Results:**

Non-irradiated controls exposed to RF and RB enhanced corneal resistance to collagenase digestion, with RB having a stronger effect than RF. RF/UV-A-treated corneas showed significantly increased digestion resistance with increasing fluence levels. RB/green-treated corneas displayed enhanced digestion resistance with each increase in fluence up to 10 J/cm²; a 15 J/cm² fluence yielded similar digestion resistance times to a 10 J/cm² fluence, suggesting a plateau effect in accelerated RB/green CXL protocols.

**Conclusions:**

When compared to standard-fluence treatments, high-fluence accelerated epi-off CXL using both riboflavin and rose bengal significantly increases resistance to enzymatic digestion. The optimal settings for clinical protocols might be 15 J/cm² (30 mW/cm² for 8 min 20 s) for RF/UV-A and 10 J/cm² (15 mW/cm² for 11 min 7 s) for RB/Green Light.

## Introduction

Corneal cross-linking (CXL) is a therapy originally used to treat corneal ectasias like keratoconus, pellucid marginal degeneration, postoperative ectasia, and keratoglobus with a number of clinical protocols varying irradiation time and fluence levels [1–7]. Subsequently, CXL indications were extended to include the treatment of infectious keratitis (IK) [2, 8–10]. 

CXL acts through the application of a chromophore to the corneal stroma and subsequent stromal irradiation with light of a specific wavelength to activate the chromophore. When riboflavin (RF) and UV light (365–370 nm) are employed, this produces in situ reactive oxygen species (ROS) that stimulate the covalent cross-linking of stromal molecules—mainly collagen fibers and proteoglycans in the extracellular matrix—resulting in corneal tissue stiffening [11].

Besides the increase in biomechanical stiffening, CXL also kills pathogens by two primary antiseptic mechanisms: ROS-mediated damage to pathogen cell membranes leading to lysis and death, and through the direct interaction of ROS and pathogen nucleic acids [12, 13]. In addition, covalently cross-linking molecules together in the stroma yields another advantage in terms of IK treatment by inducing changes in collagen molecules’ three-dimensional conformation. These conformational changes hinder collagenases from accessing their binding sites and bolster the cornea’s resistance to enzymatic digestion. This process is called steric hindrance and enables CXL to limit ulcer development and restrict the eventual corneal scar size [14–18].

Infectious keratitis (IK), a major cause of global blindness, can be attributed to several organisms including bacteria, fungi, viruses, and amoebae [19, 20]. Prompt intervention, preferably before stromal involvement and ulcer onset, greatly enhances prognosis [21]. IK often progresses rapidly, and an early onset of treatment is crucial to ensure a favorable outcome. Factors that delay appropriate treatment onset are correct identification and choice of the appropriate antimicrobial therapy, increasing antimicrobial resistance but also financial hurdles and limited access to ophthalmic care [19–24].

Today, CXL presents as a promising IK treatment strategy, especially in an era of escalating pathogen antimicrobial resistance [25]. The technique, initially explored as an adjuvant therapy for advanced ulcerative IK by Iseli et al., was later termed “photoactivated chromophore for infectious keratitis corneal cross-linking”, or PACK-CXL [8, 25]. Since 2008, PACK-CXL has been increasingly used as an adjuvant treatment in IK. In 2022, a randomized controlled phase III trial demonstrated its efficacy as first-line and standalone treatment for early to moderate bacterial or fungal keratitis [26].

Until recently, RF/UV-A PACK-CXL irradiation settings have copied the classic CXL “Dresden protocol” for keratoconus, with a UV-A fluence of 5.4 J/cm² delivered at an intensity of 3 mW/cm² for 30 min.

However, to establish RF/UV-A PACK-CXL as an alternative to antimicrobial medication, it needed to become both faster and more efficient.

Richoz and colleagues were the first to address the speed of PACK-CXL treatment by demonstrating in vitro that a delivery time of 3 instead of 30 min for the standard 5.4 J/cm² fluence would maintain the same antimicrobial killing efficacy [27]. This was the first indication that the antimicrobial killing effect of CXL might be oxygen-independent, which contrasts with the oxygen-dependent biomechanical effect of CXL for keratoconus [28]. Accelerated clinical PACK-CXL protocols were developed subsequently [9, 10, 26].

The need for an increase in efficacy of PACK-CXL protocols was addressed more recently when Kling et al. demonstrated in vitro that an increase in fluence from 5.4 to 10 and 15 J/cm² dramatically increased the PACK-CXL killing effect [29]. Initial reservations that such high fluences might in turn harm the endothelium were allayed by more recent works on the endothelial UV-A threshold level by Seiler and colleagues and the fact that customized RF/UV-A CXL for keratoconus uses similar fluence levels without consequences for the endothelium [30, 31]. Consequently, high-fluence accelerated cross-linking protocols emerged for the treatment of both ectasia and IK, almost tripling the standard 5.4 J/cm² fluence [3–5, 26, 32, 33].

While the impact of high-fluence accelerated RF/UV-A CXL and PACK-CXL protocols on biomechanical stiffening and microbial killing has been studied in detail, little is known about their effect on the cornea’s ability to resist enzymatic digestion.

CXL is not limited to the RF/UV-A light chromophore/light combination. CXL and PACK-CXL can also utilize rose bengal (RB) and 532 nm green light (RB/green) [34, 35]. Laboratory studies have shown variable pathogen susceptibility between PACK-CXL employing RF/UV-A and RB/green. Additionally, the stromal penetration depth of these chromophores differs: RF penetrates to a depth of up to 500 μm based on formulation and application, whereas RB only penetrates up to 100 μm [35–39].

The present study aims to investigate how corneal resistance to digestion is altered by accelerated high-fluence CXL protocols, and whether there is a differential digestion resistance effect between RF/UV-A and RB/green CXL.

## Materials and methods

### Specimen acquisition

Freshly enucleated porcine eyes from young adult pigs aged 6 to 8 months were obtained from a local slaughterhouse in Zurich, Switzerland, and utilized within 8 h post-enucleation.

### Study group assignment and treatment protocols

**Fig. 1:**
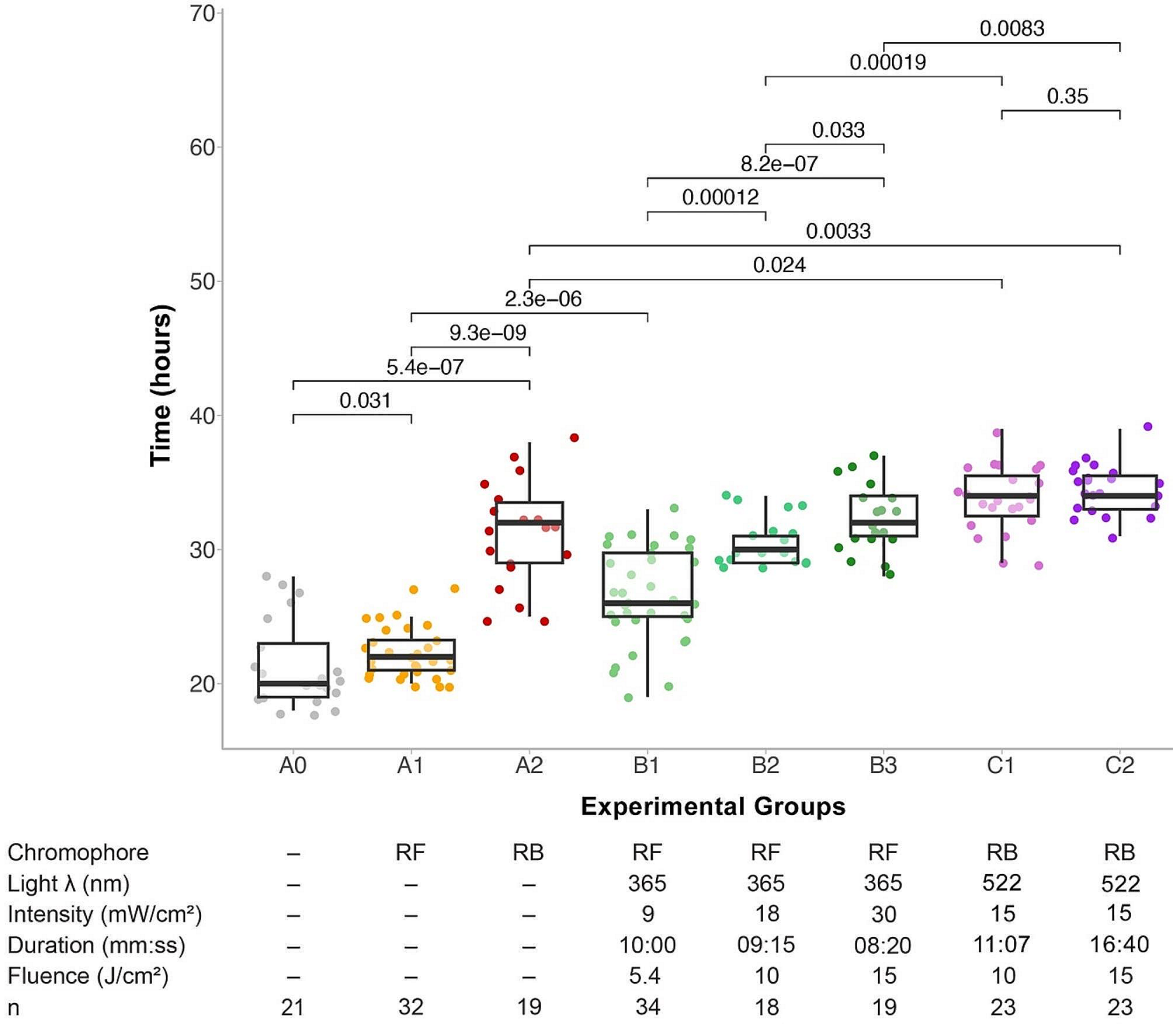
Experimental groups and experimental outcomes. **A0**: Controls, untreated. **A1**: Controls: Riboflavin soaking only, no light. **A2**: Rose Bengal soaking only, no light. **B1**: Riboflavin soaking and UV-A light, 5.4 J/cm². **B2**: Riboflavin soaking and UV-A light, 10.0 J/cm². **B3**: Riboflavin soaking and UV-A light, 15.0 J/cm². **C1**: Rose Bengal soaking and green light, 10.0 J/cm². **C2**: Rose Bengal soaking and green light, 15.0 J/cm²

The eyes were then randomly allocated to one of eight experimental groups, following distinct protocols (as detailed in Fig. 1). Each category involved unique experimental parameters including soaking solutions and varying levels of irradiation. Group A0 consisted of untreated controls. Group A1 were controls soaked with riboflavin, but not irradiated. Group A2: were controls soaked with rose bengal, but not irradiated. In group B1, corneas were soaked with riboflavin and irradiated with UV-A light at 365 nm at a fluence of 5.4 J/cm² (9 mW/cm² for 10 min). Group B2 corneas received riboflavin soaking and UV-A light at a fluence of 10.0 J/cm² (18 mW/cm² for 9 min and 15 s), whereas corneas in group B3 were soaked in riboflavin and irradiated with UV-A light at a fluence of 15.0 J/cm² (30 mW/cm² for 8 min 20 s) In group C1, corneas were soaked in rose bengal soaking and exposed to green light at 522 nm with a fluence of 10.0 J/cm² (15 mW/cm² for 11 min 7 s) In group C2, corneas were soaked in rose bengal and exposed to green light with a fluence of 15.0 J/cm² (15 mW/cm² for 16 min 40 s).

### Preparation and treatment protocols for each group

Irrespective of the group, the corneas underwent a uniform preparatory process. The epithelial layer was removed using a hockey knife, followed by circumferential excision about 3 mm from the corneoscleral rim. The excised corneas were immersed in a 400 mosmol/L PBS solution for 10 min to reach a hydration state similar to the one found in the living stroma, followed by trephination of a central 8 mm region using a biopsy punch, resulting in corneal buttons.

After receiving CXL (or, in the case of corneas in Groups A1 and A2, chromophore saturation only), all corneal buttons were rinsed with 400 mosmol/L PBS solution.

### Enzymatic digestion of corneal buttons and assessment

All corneas were transferred to a fresh 24-well plate (Merck AG, Darmstadt, Germany) with each well containing 2.0 ml of 0.3% collagenase-A solution (Roche, Basel, Switzerland). The plate was placed on a thermoshaker (37 °C with 200 revolutions per minute) and the corneal buttons were visually inspected and photographed hourly. The time until complete enzymatic digestion for each cornea was recorded. Complete digestion was defined by complete dissolution of the button and the formation of a dust-like layer.

### Statistical analysis

Statistical analysis was performed in SPSS (version 28; IBM Corporation, Armonk, New York, USA) and R Studio (version 2023.06.0). The normality of the data was verified using the Shapiro-Wilk test. Descriptive statistics were presented as mean ± standard deviation. For continuous variables, analysis of variance and the Kruskal-Wallis H test were conducted to analyze the differences between the study groups, and post-hoc tests were performed with a Bonferroni correction. A value of *P* < 0.05 was considered statistically significant for all tests.

## Results

A total of 189 corneas were assessed for their resistance to collagenase-A-mediated digestion following various interventions (Fig. 1).

### Control groups

Control groups A0 (non-irradiated, no chromophore soaking), A1 (non-irradiated, 0.1% RF soaked), and A2 (non-irradiated, 0.1% RB soaked) had mean digestion times of 21.38 ± 3.248 h, 22.31 ± 1.975 h, and 31.21 ± 3.838 h, respectively. Both RF and RB increased the resistance of corneal buttons to collagenase digestion compared with untreated, unirradiated control corneas (A1 vs. A0, *p* = 0.031; A2 vs. A0, *p* = 0.00000054). RB had a stronger inhibitory effect on digestion than RF (A2 vs. A1, *p* = 0.0000000093).

### RF/UV-A-treated corneas

RF-treated corneas that received UV-A fluences of 5.4 J/cm² (group B1), 10 J/cm² (group B2), and 15 J/cm² (group B3) demonstrated mean digestion times of 26.5 ± 3.544 h, 30.61 ± 1.787 h, and 32.32 ± 2.562 h, respectively. RF/UV-A treatment increased the resistance of corneal buttons to collagenase digestion compared with untreated, unirradiated control corneas (B1 vs. A0, *p* = 2.3 × 10^− 6^). Increasing the fluence from 5.4 J/cm² to 10 J/cm² enhanced this resistance (B2 vs. B1, *p* = 0.00012). Further increasing UV-fluence to 15 J/cm² (B3) provided a statistically significant increase in digestion time compared to both 5.4 J/cm² (B3 vs. B1, *p* = 8.2 × 10^− 7^) and 10 J/cm² (B3 vs. B2, *p* = 0.033) RF/UV-A CXL, indicating that higher fluences increase corneal digestion resistance.

### RB/green-treated corneas

RB/green-treated corneas at fluences of 10 J/cm² (C1) and 15 J/cm² (C2) presented mean digestion times of 33.7 ± 2.382 h and 34.39 ± 1.852 h, respectively. Compared with non-irradiated, 0.1% RB-soaked corneas (A2), corneas in both C1 and C2 groups showed enhanced resistance to collagenase digestion (C1 vs. A2, *p* = 0.024; C2 vs. A2, *p* = 0.0033). Increasing the fluence to 15 J/cm² (C2) resulted in similar digestion times to 10 J/cm² RB/green-treated corneas (C1; C2 vs. C1, *p* = 0.35), suggesting the presence of a plateau effect when performing accelerated RB/green CXL protocols.

## Discussion

This study examined the effects of accelerated high-fluence RF/UV-A and RB/green corneal CXL protocols on corneal resistance to enzymatic digestion. In our experiments, we specifically used fluences that are currently in clinical use in human corneas. Previous research has shown that increased corneal resistance to enzymatic digestion occurs following CXL, whether in standard fluence or accelerated high-fluence RF/UV-A CXL [26, 29, 32], or RB/green CXL [40, 41]. However, the fluence and intensities used in the previously published RB/green CXL were far beyond any clinically applied protocols, with intensities of up to 250 mW/cm² and fluences of up to 200 J/cm² [40].

In RF/UV-A-treated corneas, Group B1 (5.4 J/cm² “standard” UV-A fluence) corneas exhibited significantly greater digestion resistance compared to control groups A0 and A1. Increasing the UV-A fluence to 10 J/cm² (Group B2) further increased corneal digestion resistance compared to standard fluence (B1). Moreover, a fluence of 15 J/cm² (Group B3) significantly enhanced digestion resistance when compared to 10 J/cm² (Group B2). This might indicate a UV-A fluence dose-dependent effect without reaching a plateau. These results align with recently published research from the veterinary field focusing on (thicker) animal corneas, investigating fluences of up to 30 J/cm², which would be toxic to the human endothelium [42].

In the RB/green light-treated corneas, high-fluence accelerated CXL protocols using fluences of 10 J/cm² and 15 J/cm² provided higher enzymatic digestion resistance compared to RB alone or even high-fluence RF/UV-A CXL. This protective effect did not increase beyond the 10 J/cm² fluence, which might be due to either oxygen dependency or maximization of the steric hindrance effect on enzymatic cleavage. In 2014, Fadlallah demonstrated that RB/green CXL can in principle increase resistance to corneal digestion, but used fluences between 50 and 200 J/cm², which are far beyond what a living human cornea can tolerate in terms of endothelial light exposure [40].

Our study adds two important new aspects: (1) the inhibitory effect on digestion by RF is weaker than that of RB and (2) RF-mediated CXL reaches a peak in resistance to digestion at 15 J/cm² only, while RB-mediated CXL plateaus at 10 J/cm². However, the ideal fluence for clinical protocols will depend not only on the fluence needed for maximum enzymatic resistance effect, but also on the maximal antimicrobial effect, which is 15 J/cm² for RF-CXL and is yet to be determined for RB-PACK-CXL. It is worth noting that the maximum fluence investigated in this study, 15 J/cm², was delivered as 9 min, 15 s of 30 mW intensity irradiation. This was deliberately chosen to correspond with the prevailing maximum fluence delivered in CXL for ectasia and PACK-CXL protocols in clinical use today.

This research has several potential clinical implications. Increased digestion resistance is only one of three main actions corneal cross-linking has on the cornea, the two others being increased biomechanical stiffening (exploited in CXL for ectasia), and a direct pathogen-killing effect (exploited in PACK-CXL). Resistance to enzymatic digestion is an important feature in both applications, as it counteracts the action of proteases produced by inflammation (which can be present in ectasias) or by pathogens. Identification of CXL and PACK-CXL protocols that optimize the protease digestion resistance of the cornea, as well as provide optimal stiffening or pathogen killing-effects is therefore an important target when optimizing a corneal cross-linking protocol for a given application, especially when multiple chromophore/light combinations can be used, each with differing effects on the cornea.

### Riboflavin-mediated CXL

For pathogen killing, our research group has recently demonstrated that high-fluence RB PACK-CXL protocols are more effective than low-fluence protocols. These results indicate that, for RF/UV-A CXL, 15 J/cm²-fluence protocols should deliver an optimal pathogen-killing effect. For keratoconus, accelerated RB CXL 10 J/cm²-fluence settings deliver a good biomechanical stiffening effect and an increased enzymatic resistance effect that is superior to any of the standard (5.4 J/cm²) fluence keratoconus CXL protocols.

### Rose Bengal-mediated CXL

For RF/green light-mediated CXL, our results indicate that both 10 and 15 J/cm² accelerated protocols might be potential candidates for future clinical protocols, once the antimicrobial effect of these fluences has been assessed.

## Conclusion

The findings from our study demonstrate that both high-fluence RF/UV-A and RB/green light accelerated CXL protocols significantly enhance corneal resistance to enzymatic digestion, with RB/green CXL having a stronger effect than RF/UV-A-mediated CXL. No such limit was reached at the highest fluence of RB/UV-A CXL evaluated (15 J/cm²). Our findings may be used to optimize current clinical CXL and PACK-CXL protocols. Despite the insights gained from this research, we acknowledge its limitations, which include its *ex-vivo* nature and the use of transparent corneas.

Further research is required to fully understand the impact that accelerated high-fluence RF/UV-A and RB/green protocols, including the potential oxygen-dependency of the protective effect on enzymatic digestion, and to investigate whether simultaneous application of RF-UVA/RB-green CXL protocols may deliver additional digestion resistance benefits than either light/chromophore combination in isolation.

## Data Availability

The datasets used and/or analyzed during the current study are available from the corresponding author on reasonable request.

## Abbreviations

CXL Corneal cross: linking
PACK-CXL: Photoactivated chromophore for infectious keratitis cross-linking
RB: Rose Bengal
RF: Riboflavin
UV-A: Ultraviolet-A
